# Tracing melanin-concentrating hormone function: decades of memory research and emerging links to Alzheimer’s disease

**DOI:** 10.3389/fnins.2026.1918594

**Published:** 2026-08-24

**Authors:** Sofia Niño-Rivero, Eduardo Blanco, Valentina Rodríguez-Canclini, Laura Morelli, Patricia Lagos, Pablo Galeano

**Affiliations:** 1 Unidad Académica de Fisiología, Facultad de Medicina, Universidad de la República, Montevideo, Uruguay; 2 Departamento de Psicobiología y Metodología de las Ciencias del Comportamiento, Universidad de Málaga, Málaga, Spain; 3 Instituto de Investigación Biomédica de Málaga y Plataforma de Nanomedicina (IBIMA Plataforma BIONAND), Málaga, Spain; 4 Fundación Instituto Leloir, IIBBA-CONICET, Ciudad Autónoma de Buenos Aires, Argentina

**Keywords:** Alzheimer’s disease, melanin-concentrating hormone, memory, Neurodegeneration, sleep

## Abstract

Melanin-concentrating hormone (MCH) is a hypothalamic neuropeptide classically implicated in the regulation of energy homeostasis, sleep–wake cycle and motivated behaviors. Over the last 30 years, an increasing amount of evidence has revealed a complex, non-linear role in memory processing. In this mini-review, we summarize experimental findings on the multifaceted influence of MCH on memory, highlighting how this neuropeptide can promote consolidation by improving synaptic efficacy, induce the forgetting of hippocampus-dependent memories during REM sleep or negatively regulate memory formation. We also discuss emerging evidence linking MCH dysregulation to the pathophysiology of Alzheimer’s disease (AD). Early interest in MCH as a cerebrospinal fluid biomarker has been superseded by non-invasive blood assays. Recent data, however, suggest therapeutic potential and a unique pathological vulnerability. While emerging evidence suggests MCH somata may show early resilience, their axonal projections are severely compromised by amyloid pathology. Ultimately, tau pathology appears to drive late-stage somatic degeneration and reduced activation during REM sleep. Importantly, activating surviving MCH neurons has been shown to restore sleep architecture in preclinical models, offering a potential therapeutic target for sleep disturbances. In conclusion, decades of basic memory research together with human and preclinical studies identify the MCH system as an important neuromodulatory network for understanding the memory and sleep alterations characteristic of AD.

## 1 Introduction

Melanin-concentrating hormone (MCH) was originally isolated from teleost fish pituitary extracts (Kawauchi et al., 1983), although its synthesis takes place in hypothalamic cell bodies of the lateral nucleus lateralis tuberis (Bird et al., 1989). In the mammalian brain, this hypothalamic origin is conserved, with MCH synthesized mainly by neurons in the lateral hypothalamic area (LHA), incerto-hypothalamic area and dorsolateral zona incerta (Bittencourt et al., 1992; Diniz and Bittencourt, 2017). MCH neurons project to multiple brain areas, including the cerebral cortex, hippocampus, amygdala, nucleus accumbens, septum, locus coeruleus, raphe nuclei and other hypothalamic areas (Bittencourt et al., 1992). MCH is synthesized from a large polypeptide precursor, the prepro-MCH, and its effects are mediated by two receptors: MCHR-1 and MCHR-2 (Presse et al., 2014). MCHR-1 is expressed in rodents and humans, while MCHR-2 is absent in rodents but present in primates and some other mammals (Tan et al., 2002). MCH may also be released diffusely via ventricular volume transmission (Noble et al., 2018). MCH regulates food intake, energy balance, sleep–wake cycle, motivation, mood and reproductive behaviors (Concetti et al., 2023; Diniz and Bittencourt, 2017; MacNeil, 2013; Torterolo et al., 2011). Furthermore, the MCH system is involved in memory processes; however, the evidence is more limited with some apparently contradictory results (Adamantidis and de Lecea, 2009; Burdakov and Peleg-Raibstein, 2020, 2025; Concetti and Burdakov, 2021; Concetti et al., 2023; Izawa et al., 2019; Li et al., 2025; Ruiz-Viroga et al., 2023).

In this mini-review, we summarize three decades of evidence on the complex role of MCH in memory alongside recent findings linking its dysregulation to Alzheimer’s disease (AD), providing a unified perspective on how this neuropeptide impacts memory function and neurodegenerative decline.

## 2 Methods

We searched PubMed on February 27, 2026, and updated the search on June 15, 2026, for the terms “Melanin-concentrating hormone AND Memory” and “Melanin-concentrating hormone AND Alzheimer,” retrieving a total of 81 entries. Articles were initially screened by title and abstract, followed by a full-text review. We included 28 studies that directly assessed MCH manipulations in memory paradigms or evaluated MCH network alterations in AD patients, human tissue, or preclinical models (spanning from 1999 to 2026; Table 1). Studies were excluded if they lacked a clear link to behavioral or structural memory networks, or if they focused on physiological functions of MCH unrelated to cognition.

**Table 1:** Summary of experimental studies on MCH in memory and Alzheimer’s disease.

Reference	Model/sample	Main technical approach	Key finding
Monzon et al. (1999)	Male Wistar rat	*In vivo* pharmacology, behavioral assays	MCH enhances IA memory retrogradely
Varas et al. (2002a)	Male Wistar rat	*Ex vivo* slice electrophysiology	MCH perfusion induces NMDA-dependent potentiation of DG responses
Varas et al. (2002b)	Male Wistar rat	*In vivo* pharmacology, biochemical assays	MCH reverses L-NOArg-induced amnesia and elevates hippocampal NO/cGMP
Varas et al. (2003)	Male Wistar rats	*In vivo* pharmacology, *ex vivo* slice electrophysiology, molecular biology	MCH facilitates LTP induction and up-regulates NMDAR subunits mRNA expression in DG
Adamantidis et al. (2005)	MCHR-1^Neo/Neo^ KO mice (sex not specified)	Genetic knockout, *ex vivo* electrophysiology, behavioral assays	MCHR-1 deletion impairs inhibitory passive avoidance memory and reduced NMDA-mediated responses in CA1 *ex vivo*
Millan et al. (2008)	Male Wistar rats	*In vivo* pharmacology, microdialysis, behavioral assays	MCHR-1 blockade enhances SR and increases frontal cortex acetylcholine
Pachoud et al. (2010)	Male and female MCHR-1 KO mice	Genetic knockout, *ex vivo* electrophysiology	MCHR-1 deletion impairs CA1 hippocampal glutamatergic transmission and LTP/LTD
Le Barillier et al. (2015)	Male MCH/ataxin3 mice	Transgenic neuronal ablation, *ex vivo* electrophysiology, behavioral assays	MCH neuronal ablation impairs spatial memory and alters short-term synaptic plasticity
Chaki et al. (2015)	Male Wistar rats	*In vivo* pharmacology, behavioral assays	MCHR-1 antagonism has no effect on spatial memory in the MWM
Sita et al. (2016)	Male Wistar rats	*In vivo* pharmacology, behavioral assays	MCH neutralization in dCA3 produces no effect on working memory in a dry-land maze
Izawa et al. (2019)	Male mice (many strains)	Optogenetics, chemogenetics, *in vivo* recordings, behavioral assays	REM sleep state-dependent activation of MCH neurons promotes active forgetting of hippocampus-dependent memory
Kosse and Burdakov (2019)	Male and female mice from various transgenic lines	Fiber photometry, optogenetics, behavioral assays	MCH neuronal burst activity is required for NOR
Vawter et al. (2020)	Male and female MCHR-1 KO mice	Genetic knockout, behavioral assays	MCHR-1 deletion causes deficits in NOR, CFC, and female-specific deficit in OLR
Davis and Vanderheyden (2020)	Male long Evans rats	Optogenetics, behavioral assays	MCH-induced sleep stimulation improves fear extinction memory consolidation
Sanathara et al. (2021)	Male OXTR-cKO mice	Genetic knockout, behavioral assays	OXTR deletion in MCH neurons impairs social recognition memory
Liu et al. (2022)	Male mice	Chemogenetics, *in vivo* pharmacology, behavioral assays	MCH enhances spatial memory but reduces SR via the dCA3-dLS circuit
Mutlu-Burnaz et al. (2022)	Male mice	Chemogenetics, behavioral assays	MCH neuronal inhibition during consolidation produces no effect on NOR
Ruiz-Viroga et al. (2023)	Male Wistar rats	*In vivo* pharmacology, Western blot, behavioral assays	Intra-hippocampal MCH administration impairs memory consolidation in the NOR and mEPM tests
Harris and Burdakov (2024)	Male and female mice	Optogenetics, *ex vivo* slice electrophysiology	MCH axon activation lowers the threshold for persistent synaptic potentiation
Pham et al. (2024)	Male mice with vGlut2 ablation in MCH neurons	Conditional KO, CRISPR/Cas9 genome editing, behavioral assays	vGlut2 ablation in MCH neurons enhances object, spatial, and social memory
Schmidt et al. (2013)	Human CSF	Fluorescence immunoassay	Elevated MCH in CSF correlates with tau pathology and cognitive decline
Oh et al. (2019)	C57BL/6 J, APP/PS1 and 5XFAD mice	In vivo pharmacology, *in vitro* assays, *ex vivo* electrophysiology	Intranasal MCH reduces Aβ levels, ameliorates memory deficits and improves LTP
López-Gambero et al. (2021)	Male and female 5XFAD mice	RT-qPCR, indirect calorimetry, immunoassays	Hypothalamic *Pmch* increases alongside severe metabolic dysregulation, particularly in homozygous females
Calafate et al. (2023)	Male and female App(NL-G-F) mice/Human postmortem samples	Spatial transcriptomics, EEG/EMG, immunohistochemistry, electrophysiology	MCH axons show structural impairments, contributing to neuronal hyperactivity and sleep disturbances in early AD
Kim et al. (2024)	Male APP/PS2 mice	RNA sequencing, behavioral assays	Systemic IGF-1 treatment rescues behavioral deficits and upregulates hippocampal *Pmch* expression
Satpati et al. (2024) (preprint)	Human postmortem samples	Unbiased stereology, gene expression profiling, immunohistochemistry	Preliminary data indicate MCH somatic bodies remain structurally resilient during AD progression.
Wu et al. (2025)	Male and female Tg-SwDI mice	Sleep study (EEG/EMG), immunohistochemistry	Number and size of MCH neurons remain unchanged while orexin neurons degenerate.
Pang et al. (2026) (preprint)	Male and female PS19, hTau and mutant P301L-tau mice	Longitudinal EEG/EMG, in vivo fiber photometry, optogenetics, chemogenetics	Preprint data suggest late-stage tauopathy causes significant MCH somatic loss and depresses REM-associated calcium dynamics. Chemogenetic and optogenetic activation of surviving MCH neurons was reported to restore sleep architecture.

## 3 Results

### 3.1 Experimental evidence on the role of the MCH in memory processing

In this section, we review the literature investigating how the MCH system modulates memory. To navigate the complexity of the reported findings, the following subsections categorize the evidence based on the functional outcome of MCH signaling: facilitating memory, inducing active forgetting, impairing memory formation, or reporting null effects.

#### 3.1.1 Evidence supporting necessary and facilitatory roles of MCH-related signaling in memory

Here, we discuss the substantial body of evidence indicating that MCH signaling is necessary for, or actively facilitates, memory consolidation and synaptic plasticity. This literature, spanning from early pharmacological studies to recent optogenetic circuit manipulations, is grouped by the primary experimental methodology used to uncover these effects.

##### 3.1.1.1 Pharmacological and *ex vivo* MCH administration

Pioneering work by De Barioglio’s group first established that MCH facilitates memory consolidation. Monzon et al. (1999) found that MCH administration into the hippocampus or amygdala immediately after training retrogradely enhances inhibitory avoidance (IA) memory, a one-trial learning task where an animal avoids stepping down from a safe platform, having learned from a single prior training event that the grid below delivers an aversive foot shock. Electrophysiological *ex vivo* experiments performed on hippocampal slices revealed that perfusing the slices with MCH induces a long-lasting potentiation of dentate gyrus (DG) evoked responses. This synaptic plasticity depends on interactions with the N-methyl-D-aspartate (NMDA) receptor (NMDAR) as it was blocked by dizocilpine (MK-801) but not by 2-amino-5-phosphonovaleric acid (APV) (Varas et al., 2002a). The group then showed that intra-hippocampal injection of MCH *in vivo* increases susceptibility to *ex vivo* induction of long-term potentiation (LTP) and up-regulates NMDAR subunit mRNA expression in the DG (Varas et al., 2003). Finally, they also showed that the nitric oxide (NO) cascade, a key retrograde signaling pathway in the hippocampus that facilitates synaptic plasticity and memory consolidation (Hölscher, 1997), plays a critical role, since intra-hippocampal or intra-amygdalar MCH administration reversed the amnesic effect induced by the NO synthase inhibitor NG-nitro-L-arginine (L-NOArg) and elevated hippocampal NO and cyclic guanosine monophosphate (cGMP) levels (Varas et al., 2002b). These results strongly suggest that MCH improves memory consolidation by enhancing hippocampal synaptic efficacy through NMDAR-mediated mechanisms and NO/cGMP signaling.

##### 3.1.1.2 Genetic deletion of MCHR-1 or specific genes within MCH neurons

MCHR-1 knockout (KO) mice exhibit deficits in inhibitory passive avoidance (Adamantidis et al., 2005). They also show impairments in novel object recognition (NOR, a task that evaluates recognition memory by measuring the ability of the animal to discriminate between a familiar object and a novel one, given the innate preference of rodents for exploring novelty), contextual fear conditioning (CFC, a paradigm assessing associative memory by pairing a specific environment with an aversive foot shock), and a female-specific impairment in the object location recognition (OLR, a task that evaluates spatial memory based on the natural tendency of rodents to preferentially explore an object that has been moved to a novel location compared to one remaining in its familiar position) (Vawter et al., 2020). *Ex vivo* electrophysiological experiments have demonstrated that these behavioral deficits are associated with alterations in hippocampal glutamatergic signaling. Specifically, recordings from hippocampal slice preparations revealed significantly reduced NMDA receptor-mediated responses in MCHR-1 KO mice, reductions in both AMPA- and NMDA-mediated glutamatergic transmission, lower amplitudes of miniature excitatory postsynaptic currents and NMDA/AMPA ratios, and deficits in LTP and long-term depression (LTD). Furthermore, quantitative *in situ* hybridization demonstrated a decrease in NMDAR-1 subunit mRNA expression in the *Cornu Ammonis* 1 (CA1) region (Adamantidis et al., 2005; Pachoud et al., 2010). Importantly, the LTP deficit in the MCHR-1 KO mice is attributable to an impairment in induction mechanisms, since repeated high-frequency stimuli successfully restore potentiation to control levels (Pachoud et al., 2010).

In a more recent study by Sanathara et al. (2021), it was shown that oxytocin-MCH signaling also modulates social memory, since the selective deletion of oxytocin receptors (OXTR) in MCH neurons impairs social recognition (SR) memory (the ability of an animal to identify and remember a specific conspecific) but not general sociability, which is the basic innate motivation to engage in social interaction. In addition, MCH neurons in these OXTR-cKO mice show reduced direct presynaptic inputs from the paraventricular nucleus, lateral hypothalamus, nucleus accumbens and ventral tegmental area, suggesting that oxytocin signaling regulates the functional connectivity of MCH neurons.

In addition to neuropeptide release, MCH neurons exhibit a glutamatergic and GABAergic neurochemical phenotype (Jego et al., 2013; Chee et al., 2015). To examine the role of glutamatergic signaling from MCH neurons in memory processes, Pham et al. (2024) ablated the *Slc17a6* gene (encoding the vesicular glutamate transporter 2 (VGLUT2) transporter) in MCH neurons by using two approaches: a conditional KO model based on the Cre/loxP system and an MCH neuron-specific *in vivo* CRISPR/Cas9 genome-editing approach in adult mice. This complementary approach was utilized because standard Cre/loxP systems typically induce gene excision during embryogenesis, introducing the risk of compensatory developmental plasticity. Conversely, the *in vivo* CRISPR/Cas9 intervention enables acute, temporally controlled gene silencing in mature neural circuits, effectively precluding these early developmental confounds. Confirming that the memory enhancement was not a developmental artifact, increased performance in the NOR task was observed in both genetic models, while in the CRISPR/Cas9 model an enhanced performance in the OLR and SR tasks was also observed. These findings suggest that glutamatergic signaling from MCH neurons participates in the regulation of object, spatial and social recognition memory.

##### 3.1.1.3 MCH neuronal ablation model

To avoid the neurodevelopmental confounds inherent to KO models, Le Barillier et al. (2015) utilized an MCH/ataxin3 transgenic mouse model to progressively and selectively ablate MCH neurons after birth. MCH/ataxin3 mice exhibited deficits in several hippocampal-dependent forms of memory. These included impaired short-term spatial memory in a delayed non-match-to-sample T-maze (where animals must recall a previously visited arm and choose the alternate one) and slower acquisition of spatial reference memory, the ability to learn and remember a fixed location across repeated trials, in the Morris water maze (MWM), a navigation task requiring rodents to use environmental cues to locate a hidden submerged platform. Moreover, MCH/ataxin3 mice did not habituate normally to a novel environment in the open field (OF) test, an assay where habituation is measured as the natural decrease in exploration over time as the animal learns the environment is familiar. Electrophysiological analyses showed that early phases of both LTP and LTD were impaired at Schaffer collateral-CA1 synapses. These early phase deficits were associated with altered hippocampal short-term synaptic plasticity, specifically decreased post-tetanic potentiation and increased synaptic depression.

##### 3.1.1.4 Optogenetic and chemogenetic circuit manipulations

Recent applications of optogenetics, chemogenetics and *in vivo* photometry approaches have helped to determine the circuit-specific dynamics of MCH signaling in memory processing. Kosse and Burdakov (2019) demonstrated via fiber photometry that lateral hypothalamic MCH neurons display bursts of activity during object encounters, with these bursts being more intense during the exploration of new objects and decreasing as the objects become familiar. They also demonstrated that the optogenetic inhibition of MCH neurons when animals encountered objects during the training trial in the NOR task blocked the preference for the novel object during the test trial. This memory formation is controlled by a local inhibitory microcircuit of neighboring GABAergic neurons (GAD65+). Chemogenetic activation of these neurons suppresses the MCH cell bursts associated with novel object encounters, while optogenetic silencing of these neurons increases performance in the NOR task. This effect is mediated by MCH signaling, as it is fully blocked by the MCH receptor antagonist SNAP94847.

Other studies have demonstrated that MCH modulates memory by acting in extra-hypothalamic circuits. Liu et al. (2022) showed that MCH modulates the flow of information from the dorsal *Cornu Ammonis* 3 (dCA3) region of the hippocampus to the dorsolateral septum (dLS). They found that MCH acts pre-synaptically to enhance the dCA3 excitatory input strength and GABA release, which increases the overall GABA tone and attenuates feed-forward inhibition. In this manner, MCH synchronizes dLS neuronal firing with their input from dCA3. Behaviorally, enhancing MCH signaling via direct pharmacological infusion of an agonist into the dLS or chemogenetic activation of dLS-projecting MCH neurons improved spatial memory in the MWM. Moreover, the pharmacological activation of MCHR-1 in the dLS increased attention to environmental cues in the OF test and decreased SR. In contrast, MCH signaling blockade, either via direct infusion of an MCHR-1 antagonist into the dLS or via chemogenetic inhibition of these projection neurons, impaired spatial memory. In line with these extra-hypothalamic effects, optogenetic activation of MCH axons in hippocampal slices lowers the threshold for long-lasting potentiation (Harris and Burdakov, 2024).

Finally, Davis and Vanderheyden (2020) tested in a single prolonged stress rat model of post-traumatic stress disorder (PTSD) whether optogenetic enhancement of post-trauma sleep via activation of MCH neurons could rescue fear-associated memory impairments. Physiologically, optogenetic stimulation increased rapid eye movement (REM) sleep duration in both the light and dark phases, as well as non-rapid eye movement sleep (NREM) sleep duration specifically in the dark phase. Behaviorally, rats that received optogenetic stimulation of MCH neurons for seven consecutive days following trauma did not differ from non-stimulated animals in terms of freezing duration during the initial fear conditioning or extinction acquisition phases (the process where an animal repeatedly experiences the conditioned context without the shock, gradually learning it is no longer a threat). However, 24 h after extinction, during the fear extinction recall test, a measure of how well animals remember that a previously aversive context is no longer threatening, the stimulated rats demonstrated significantly less freezing behavior. This suggests that sleep enhancement induced by MCH is associated with improved extinction memory consolidation, which rescues the fear extinction deficit characteristic of this PTSD model.

#### 3.1.2 Evidence supporting a role of MCH system in active forgetting

A paradigm-shifting study by Izawa et al. (2019) demonstrated a critical role of the MCH in the active forgetting of hippocampus-dependent memories. These are memories that rely on the structural and functional integrity of the hippocampal formation for proper encoding, consolidation, and retrieval (Broadbent et al., 2004; Kim and Fanselow, 1992; Morris et al., 1982). Specifically, the processing of spatial navigation, contextual fear associations, and object recognition information required to successfully perform tasks such as the MWM, CFC, and NOR heavily engages this circuitry (Broadbent et al., 2004; Kim and Fanselow, 1992; Morris et al., 1982). Using optogenetic and chemogenetic approaches, Izawa et al. (2019) showed that post-training activation of lateral hypothalamic MCH neurons impairs retention in the NOR and CFC tasks, whereas their inhibition or temporally-controlled genetic ablation improves performance in these two behavioral paradigms and in the MWM. This active forgetting is mediated by MCH projections to the dorsal CA1 (dCA1) region, since optogenetic stimulation of these inputs is sufficient to disrupt the performance in the NOR and CFC tasks. Mechanistically, electrophysiological recordings revealed that activation of the dCA1-projecting MCH fibers inhibits the activity of hippocampal pyramidal neurons by potentiating local inhibitory transmission. Furthermore, fiber photometry and single-cell microendoscopy revealed that wake-active and REM sleep-active MCH neurons are actually distinct subpopulations. Restricting the selective optogenetic inhibition of MCH neurons exclusively to REM sleep episodes improved memory performance in the NOR task. This study demonstrates that REM sleep-active MCH circuits actively suppress hippocampus-dependent memories through targeted inhibitory modulation of dCA1.

#### 3.1.3 Evidence supporting a negative regulation of memory by MCH-related signaling

A distinct body of evidence indicates MCH signaling can impair memory. Millan et al. (2008) showed that systemic administration of MCHR-1 antagonists (SNAP-7941 and GW3430) reverses scopolamine-induced SR deficits and mitigates spontaneous forgetting, while *in vivo* microdialysis experiments showed that administration of both antagonists increased extracellular acetylcholine levels in the frontal cortex. Thus, the pro-cognitive effects observed following MCHR-1 antagonism support the hypothesis that endogenous MCH signaling could have a detrimental effect on memory processes through modulation of cortical cholinergic neurotransmission. Consistent with these results, Liu et al. (2022), as previously described, showed that activating MCH receptors pharmacologically in the dLS decreases SR.

In a more recent study by our group (Ruiz-Viroga et al., 2023), we administered MCH bilaterally into the hippocampus immediately after training and assessed its effects using two hippocampus-dependent behavioral paradigms: the NOR and the modified Elevated Plus Maze (mEPM) tasks. In memory paradigms, the mEPM evaluates learning and retention by measuring the latency of an animal to move from an exposed open arm to a safer enclosed arm across repeated trials. While lower doses (25 and 50 ng) produced no behavioral effect, higher doses of MCH (200 and 500 ng) impaired performance in both tasks, indicating deficits in object and spatial memory consolidation, while the MCHR-1 antagonist ATC-0175 prevented the MCH-induced impairment in the NOR, suggesting a dependence on MCHR-1. Additionally, Western blot analyses revealed reduced MCHR-1 and tropomyosin receptor kinase B (TrkB) protein expression in the hippocampus following MCH administration. As the high-affinity receptor tyrosine kinase for brain-derived neurotrophic factor (BDNF), TrkB is biologically essential for triggering the intracellular signaling networks that govern synaptic plasticity and the consolidation of new memories (Bekinschtein et al., 2014; Reichardt, 2006). Because the expression levels of both MCHR-1 and TrkB receptors correlated positively with performance in the NOR task, these results suggest that MCH negatively regulates hippocampal-dependent memory consolidation, potentially through receptor co-internalization and degradation, thereby impairing the BDNF/TrkB signaling cascade necessary for proper recognition memory formation (Ruiz-Viroga et al., 2023).

#### 3.1.4 Studies reporting null effects of MCH-related manipulations on memory

While the previous sections outline a clear role for MCH in memory processing, several studies have reported null effects. For instance, pre-training oral administration of the MCHR-1 antagonists TASP0382650 and TASP0489838 failed to impair or improve spatial learning in the MWM (Chaki et al., 2015). In a different spatial navigation paradigm, neutralizing MCH signaling directly within the *Cornu Ammonis* 3 (CA3) via bilateral anti-MCH antibody microinfusions had no impact on working memory, the temporary storage of trial-specific information needed to complete a task, in an appetitive dry-land maze, which assesses spatial navigation without the stress associated with water-based tasks (Sita et al., 2016). Null effects have also been observed in genetic models, as Vawter et al. (2020) demonstrated that male MCHR-1 KO mice show unimpaired spatial memory in the OLR task. Furthermore, transiently inhibiting MCH neuronal activity during the post-training consolidation window produced no measurable effect on the NOR task (Mutlu-Burnaz et al., 2022). These negative findings highlight the biological complexity of MCH signaling, cautioning against broad generalizations.

### 3.2 Participation of MCH in Alzheimer’s disease

Alzheimer’s disease (AD) is a multifactorial disorder clinically characterized by progressive cognitive and functional decline, and neuropathologically defined by amyloid-*β* (Aβ) plaques, tau tangles, neuroinflammation and widespread metabolic and structural brain abnormalities, being hippocampus and cortex the most affected areas (Livingston et al., 2020; DeTure and Dickson, 2019; Kumar et al., 2024; Campanelli et al., 2025). In addition, multiple neurotransmission systems are severely altered in AD (Akyuz et al., 2025). Furthermore, a number of recent studies have shown a direct link between MCH signaling and AD (Calafate et al., 2023; Kim et al., 2024; López-Gambero et al., 2021; Oh et al., 2019; Satpati et al., 2024; Schmidt et al., 2013; Wu et al., 2025). Next, we discuss this emerging evidence to reveal how modifications in the MCH network improve our understanding of AD progression, focusing first on its clinical biomarker utility and therapeutic potential, and then on the specific vulnerabilities of MCH circuits during neurodegeneration.

#### 3.2.1 Clinical biomarker utility and therapeutic potential of MCH

Clinical observations first prompted interest in the MCH system regarding AD. In 2013, Schmidt et al. found that the MCH levels in cerebrospinal fluid (CSF) in AD patients were higher than those in healthy controls, positively correlated with tau pathology, and negatively correlated with cognitive performance. However, the emergence of highly accurate blood-based biomarkers, specifically plasma phosphorylated tau (such as p-tau217) and the amyloid-β (Aβ) 42/40 ratio, makes it unlikely that MCH will be developed as a biomarker for AD. As demonstrated by Palmqvist et al. (2024), these blood biomarkers have a transformative relevance for AD diagnosis, providing exceptionally high accuracy for detecting the disease in both primary and secondary care settings. They offer a scalable, minimally invasive tool that directly identifies core AD neuropathology. In contrast, evaluating MCH relies on invasive lumbar punctures for CSF extraction, and its alterations likely reflect downstream dysregulation of lateral hypothalamic circuits rather than a primary diagnostic driver of the disease. Therefore, while MCH remains a valuable target for understanding AD pathophysiology, its utility as a clinical biomarker is superseded by these accessible blood assays.

While its diagnostic value is limited, the MCH system has garnered interest from a therapeutic perspective. Based on this potential, Oh et al. (2019) demonstrated that exogenous intranasal MCH administration ameliorated memory impairments in animal models of AD. For instance, a short-term, 7-day daily intranasal MCH regimen reversed memory retention deficits induced by direct intracerebroventricular Aβ1-40 injections in C57BL/6 J mice. Likewise, in the APP/PS1 transgenic line, a model characterized by robust amyloidosis, chronic intranasal MCH treatment (administered three times a week for 3 months) successfully rescued similar memory impairments. In both models, performance was measured using the passive avoidance task, a paradigm that assesses associative learning and memory retention by evaluating the ability of the animal to remember an aversive foot shock and consequently suppress its natural instinct to enter a preferred dark compartment. In addition, in the APP/PS1 transgenic mice the chronic treatment with MCH reduced the accumulation of soluble Aβ1-42 in the cerebral cortex and upregulated key synaptic markers [BDNF, TrkB and postsynaptic density protein 95 (PSD95)] in both the cerebral cortex and hippocampus. Furthermore, *in vitro* assays utilizing SH-SY5Y human neuroblastoma cells and primary cortical neurons isolated from C57BL/6 J mouse embryos demonstrated that MCH inhibits cytotoxicity induced by Aβ. Alongside these findings, *ex vivo* electrophysiological recordings from acute hippocampal slices revealed that MCH improves LTP in the CA1 region of 5XFAD mice, an aggressive transgenic AD model which drives rapid amyloid plaque deposition (Oakley et al., 2006). In addition to these findings, systemic administration of insulin-like growth factor 1 (IGF-1) significantly ameliorated anxiety/obsessive-compulsive and depressive/motivational behaviors, as evaluated via marble burying and nestlet shredding tests, in the APP/PS2 mouse model of AD. Notably, total RNA sequencing of whole hippocampal tissue extracts revealed that this IGF-1-mediated behavioral recovery was accompanied by a significant upregulation of prepro-melanin-concentrating hormone (*Pmch*) gene expression in the hippocampus, pointing to a potential association between peripheral IGF-1-mediated behavioral recovery and hippocampal MCH pathway modulation, though the precise mechanistic link warrants further investigation (Kim et al., 2024).

#### 3.2.2 Hypothalamic vulnerability: early axonal dysfunction, transcriptional alterations and potential late-stage somatic degeneration

AD progression increasingly disrupts LHA networks. In a recent preprint report, Satpati et al. (2024), alongside peer-reviewed findings by Wu et al. (2025), described a substantial loss of orexin neurons in postmortem human brains across progressive Braak stages and in Tg-SwDI mice, respectively. On the contrary, the somata of adjacent MCH neurons appear to remain relatively spared even in advanced human Braak stages. Despite this somatic resilience, their functional integrity is compromised early on. Even when MCH cell bodies survive, their axonal projections and synaptic functions are severely compromised by amyloid pathology. For instance, in App^NL-G-F^ mice, MCH axons projecting to the CA1 region show progressive structural abnormalities, including enlarged boutons and axonal spheroids adjacent to Aβ plaques, a phenotype also observed in the human AD hippocampus (Calafate et al., 2023). Given that MCH downregulates CA1 excitatory transmission, these structural defects in MCH-dependent synapses fail to stabilize CA1 excitability, contributing to abnormal hippocampal hyperactivity and increased seizure susceptibility. Importantly, a causal role for MCH signaling in maintaining hippocampal stability is supported by the finding that exogenous MCH administration is sufficient to normalize this altered excitatory drive. Furthermore, these structural and functional abnormalities are accompanied by system-wide disruptions, as a decreased fraction of active MCH neurons directly correlates with a reduction in REM sleep in this model. Additionally, early transcriptional shifts occur, such as increased hypothalamic *Pmch* mRNA expression in 6-month-old 5XFAD mice exhibiting metabolic dysregulation (López-Gambero et al., 2021).

Ultimately, somatic resilience declines in aggressive tauopathy models, as a recent preprint report indicates that PS19 mice show degeneration of both MCH and orexin neurons (Pang et al., 2026). While both populations are structurally affected, preliminary data suggest MCH neurons exhibit selective functional impairment, specifically in their firing rate during REM sleep, which may reflect a direct impact of pathological tau on homeostasis rather than non-specific consequences of global P301L-tau overexpression. Supporting this, preliminary data suggest that cell-autonomous mutant tau expression in MCH neurons sufficiently recapitulates both neuronal loss and sleep fragmentation (Pang et al., 2026). Interestingly, chemogenetic and optogenetic activation of surviving MCH neurons was found to restore sleep architecture despite advanced neurodegeneration, highlighting a potentially tractable therapeutic target (Pang et al., 2026). Together, these findings demonstrate that structural and functional pathology in the MCH system spans a progressive spectrum from early axonal disconnection to late somatic vulnerability in AD.

## 4 Conclusion

Research over the past 30 years has shown MCH to be a complex, state- and circuit-dependent modulator of memory. A comprehensive review of the literature reveals no definitive pattern to suggest that the observed differences in MCH function (whether facilitating memory, inducing active forgetting, impairing memory formation, or producing null effects) are strictly due to the species used (rats vs. mice) or general experimental conditions. However, the emotional valence of the specific behavioral paradigm employed appears to play a critical role. For instance, an apparent contradiction exists within the acute pharmacological literature, as immediate post-training intra-hippocampal MCH administration facilitates inhibitory avoidance memory (Monzon et al., 1999; Varas et al., 2002b, 2003) but impairs consolidation in the NOR and mEPM tasks (Ruiz-Viroga et al., 2023). Because the effective doses across these studies were identical, this divergence likely stems from the nature of the tasks. This suggests MCH may facilitate the consolidation of highly aversive, threat-associated memories while impairing the consolidation of non-threatening, exploratory information. Beyond task valence, it is important to note a methodological bias in the current literature: a significant proportion of acute intervention studies have specifically targeted the post-training consolidation window, while the acquisition and retrieval phases remain comparatively underexplored. While this bias limits our complete understanding of MCH across all memory stages, the apparent duality of its effects can currently be best reconciled by considering both the emotional valence of the tasks and the highly specific neural circuits engaged. For instance, MCH projections to the dCA1 promote active forgetting via local inhibitory transmission. Crucially, active forgetting in a healthy brain is not a functional failure, but rather an essential adaptive process required for optimal memory function, as it clears overloaded or unnecessary information to maintain cognitive balance. Conversely, MCH signaling in the dCA3-dLS circuit enhances spatial memory. While some results still require independent replication, the functional outcome of MCH modulation appears to rely heavily on the specific brain region, the neural network engaged, and the emotional valence of the learning experience.

This physiological complexity provides a critical framework for interpreting the therapeutic potential of MCH in AD. The apparent contrast between the specific instances where MCH impairs memory formation or induces active forgetting in healthy animals, and the MCH-mediated memory improvement observed in AD models, is likely driven by profound differences in disease state and sleep regulation, as well as distinct experimental treatment regimens. While studies in healthy animals typically employ acute interventions to dissect specific memory phases, the memory benefits reported in AD models have been achieved through subchronic or chronic MCH administration. However, this acute versus chronic explanatory framework remains a post-hoc synthesis. A critical experimental gap exists, as the *in vivo* behavioral effects of acute MCH administration in AD models, or chronic MCH administration in healthy, intact animals, have not been systematically evaluated. Future studies must directly test these crossover regimens to determine if the therapeutic benefits in AD are strictly dependent on chronic dosing protocols or if they reflect an inherent state-dependent shift in MCH network functionality.

The MCH network exhibits a progressive, stage-dependent vulnerability to AD pathology, characterized by early-stage axonal dysfunction and what emerging evidence suggests may be late-stage somatic degeneration. This progressive deterioration provides a direct mechanistic explanation for both the aberrant sleep architecture and the hippocampal hyperexcitability observed in AD models. Specifically, structural damage to MCH projections impairs their normal ability to downregulate CA1 excitatory transmission. This failure to stabilize excitability, combined with a severe loss of MCH firing during REM sleep, deeply disturbs neuronal homeostasis. In this severely compromised neurodegenerative context, exogenous MCH administration or targeted activation of surviving MCH neurons may act as a restorative agent. Rather than impairing memory formation or inducing active forgetting, effects observed under specific acute conditions in healthy, intact circuits, a sustained restoration of MCH tone normalizes excitatory drive and appears to rescue sleep architecture, providing a net systemic benefit that may ameliorate memory decline. Crucially, because surviving MCH neurons remain functionally capable of inducing sleep despite ongoing neurodegeneration, they represent a highly appealing and functionally tractable target for therapy.

Future studies should investigate independent validation of the therapeutic potential of MCH in diverse preclinical models. Importantly, most of the evidence obtained came from preclinical models that recapitulate familial AD or aggressive tauopathies, and therefore it is not clear to what extent these findings can be extrapolated to sporadic AD. Furthermore, given that MCHergic alterations have been observed in other amyloidogenic neurodegenerative diseases like Parkinson’s disease (Oh et al., 2024; Thannickal et al., 2007), future studies should explore if this system represents a shared vulnerability across a broader pathological spectrum. Understanding these dynamics may ultimately lead to new neuropeptide-targeted therapeutic strategies for ameliorating both memory impairment and aberrant sleep phenotypes in AD, while opening new treatment avenues for related neurodegenerative disorders.

## Glossary

A*β*: Amyloid-*β*
AD: Alzheimer’s disease
AMPA: *α*-amino-3-hydroxy-5-methyl-4-isoxazolepropionic acid receptor
APV: 2-amino-5-phosphonovaleric acid
BDNF: Brain-derived neurotrophic factor
CA1: *Cornu Ammonis* 1 region of the hippocampus
CA3: *Cornu Ammonis* 3 region of the hippocampus
CFC: Contextual fear conditioning
cGMP: Cyclic guanosine monophosphate
CSF: Cerebrospinal fluid
dCA1: Dorsal CA1 region
dCA3: Dorsal CA3 region
DG: Dentate gyrus
dLS: Dorsolateral septum
GABA: Gamma-aminobutyric acid
GAD65: Glutamic acid decarboxylase 65
IA: Inhibitory avoidance
IGF-1: Insulin-like growth factor 1
KO: Knockout
L-NOArg: Nitric oxide synthase inhibitor NG-nitro-L-arginine
LHA: Lateral hypothalamic area
LTD: Long-term depression
LTP: Long-term potentiation
MCH: Melanin-concentrating hormone
MCHR-1: Melanin-concentrating hormone receptor 1
MCHR-2: Melanin-concentrating hormone receptor 2
mEPM: Modified Elevated Plus Maze
MK-801: Dizocilpine
MWM: Morris water maze
NMDA: N-methyl-D-aspartate
NMDAR: NMDA receptors
NO: Nitric oxide
NOR: Novel object recognition
NREM: Non-rapid eye movement sleep
OF: Open field
OLR: Object location recognition
OXTR: Oxytocin receptor
*Pmch*: Prepro-melanin-concentrating hormone gene
PMCH: Prepro-melanin-concentrating hormone protein
PSD95: Postsynaptic density protein 95
PTSD: Post-traumatic stress disorder
REM: Rapid eye movement sleep
SR: Social recognition
TrkB: Tropomyosin receptor kinase B
vGlut2: Vesicular glutamate transporter 2
